# Merkel cell polyomavirus small tumour antigen activates the p38 MAPK pathway to enhance cellular motility

**DOI:** 10.1042/BCJ20200399

**Published:** 2020-07-31

**Authors:** Samuel J. Dobson, Anthony Anene, James R. Boyne, Jamel Mankouri, Andrew Macdonald, Adrian Whitehouse

**Affiliations:** 1 School of Molecular and Cellular Biology, University of Leeds, Leeds LS2 9JT, U.K.; 2 Astbury Centre for Structural Molecular Biology, University of Leeds, Leeds LS2 9JT, U.K.; 3 Centre for Cancer Genomics and Computational Biology, Barts Cancer Institute, Queen Mary University of London, Charterhouse Square, London EC1 M 6BQ, U.K.; 4 School of Applied Sciences, University of Huddersfield, Queensgate, Huddersfield HD1 3DH, U.K.

**Keywords:** Merkel cell carcinoma, Merle cell polyomavirus, p38 MAPK

## Abstract

Merkel cell carcinoma (MCC) is an aggressive skin cancer with high rates of recurrence and metastasis. Merkel cell polyomavirus (MCPyV) is associated with the majority of MCC cases. MCPyV-induced tumourigenesis is largely dependent on the expression of the small tumour antigen (ST). Recent findings implicate MCPyV ST expression in the highly metastatic nature of MCC by promoting cell motility and migration, through differential expression of cellular proteins that lead to microtubule destabilisation, filopodium formation and breakdown of cell–cell junctions. However, the molecular mechanisms which dysregulate these cellular processes are yet to be fully elucidated. Here, we demonstrate that MCPyV ST expression activates p38 MAPK signalling to drive cell migration and motility. Notably, MCPyV ST-mediated p38 MAPK signalling occurs through MKK4, as opposed to the canonical MKK3/6 signalling pathway. In addition, our results indicate that an interaction between MCPyV ST and the cellular phospatase subunit PP4C is essential for its effect on p38 MAPK signalling. These results provide novel opportunities for the treatment of metastatic MCC given the intense interest in p38 MAPK inhibitors as therapeutic agents.

## Introduction

Merkel cell carcinoma (MCC) is a rare but highly aggressive neuroendocrine cancer of the skin [1]. Despite its rarity, the number of MCC cases is increasing due to greater UV exposure and immunosuppression, owing to the increased use of immunoregulatory drugs and an ageing population [2]. MCC is a highly metastatic cancer, with a high probability of regional and/or distal recurrence, contributing to the poorest 5-year survival rates of any skin cancer type [3]. Caucasian populations are particularly at risk, with a positive correlation between geographic UVB radiation indices and onset of disease [4]. It is reported that >80% of all MCCs are associated with Merkel cell polyomavirus (MCPyV), whilst the remaining MCPyV-negative MCCs display substantial C to T single nucleotide somatic mutations, consistent with UV-induced genotoxicity [5,6].

MCPyV maintains a persistent, asymptomatic infection that is acquired during childhood [7]. Like all polyomaviruses, MCPyV contains a small circular double-stranded DNA genome that sequentially expresses early tumour antigens prior to genome replication and the expression of structural proteins. Oncogenic transformation requires UV-mediated mutations that lead to truncation of the large tumour antigen (LT) and its integration into the host genome [5,8,9]. This truncation leads to a loss of the helicase binding region within LT that is required for the initiation of genome replication and late protein production, thereby stalling the viral lifecycle in a state primed for viral replication [10]. In all sequenced MCPyV-positive MCCs, truncated LT (tLT) retains the region responsible for binding to the retinoblastoma protein [11,12]. Whilst LT is truncated, the small tumour antigen (ST) retains its full length, and despite the expression of both tLT and ST being essential for MCC cell survival and proliferation, ST is the major oncogene responsible for MCPyV-induced MCC [13,14]. MCPyV ST induces the hyperphosphorylation of 4E-BP1, which facilitates the dysregulation of cap-dependent translation and restricts the ability of the E3 ubiquitin ligase SCF^Fbw7^ to degrade LT. ST also interacts with MYCL and the Ep400 chromatin remodelling complex to manipulate gene expression to promote glycolytic metabolism [13,15,16].

The prototypic polyomavirus simian virus 40 (SV40) induces transformation thorough its interaction with protein phosphatase PP2A Aα [17,18]. In contrast, MCPyV ST induces anchorage- and contact-independent growth in rodent cell lines, independent of LT or PP2A Aα [19]. MCPyV ST interacts with the PP4C–PP4R1 complex to act as a bridge to the adaptor protein, NEMO, which deactivates NF-κB signalling and subsequent pro-inflammatory cytokine production [20,21]. The MCPyV ST interaction with PP4C also leads to microtubule destabilisation, remodelling of the actin cytoskeleton and disruption of the integrity of cell–cell junctions, driving cell migration [22]. The interaction of MCPyV ST with PP4C is also required for dephosphorylation of β1 integrins to activate Rho-GTPases, specifically cdc42 and RhoA, known to enhance cell motility and migration [23].

Cell motility and migration are complex processes that predispose metastasis [24], to which the manipulation and rearrangement of the cytoskeleton is essential [25,26]. Mitogen-activated protein kinases (MAPKs) are essential regulators of complex and multi-tiered signalling cascades that are activated by a multitude of extracellular stress stimuli including UV exposure, mitogens, osmotic stress and pro-inflammatory cytokines. MAPK activation enhances cell proliferation, gene expression, cell motility and cell survival dependent on cell type [27–30]. Classical MAPKs are subdivided into extracellular receptor kinases (ERKs), p38 MAPKs, c-Jun N-terminal kinases (JNKs) and ERK5, also termed big MAPK-1 (BMK1) [31,32].

The role of p38 MAPK signalling in disease states is both complex and tissue specific [33]. Activated p38 MAPK signalling is pro-oncogenic in skin, brain, lung, ovarian, prostate and bladder cancers [34–41]. These effects are mediated through the ability of p38 MAPK to promote transcriptional activity and MAPKAPK2 (MK2) signalling, which enhances actin polymerisation through phosphorylation mediated positive end-dissociation, with known roles in mRNA stabilisation [42–46]. Despite the dysregulation of p38 in disease states, the mechanisms through which phenotypic changes occur remain poorly defined.

Here, we demonstrate that expression of MCPyV ST leads to activation of the p38 pathway to drive cellular migration and motility, with p38 inhibition sufficient to ablate motile phenotypes. We further show that the interaction between MCPyV ST and PP4C is essential for pathway activation. Given intense pharmaceutical interest in p38 pathway inhibition, there is future potential that suitable therapeutics could be repurposed to restrict the highly metastatic nature of MCPyV-positive MCCs.

## Materials and methods

### Plasmids, antibodies and inhibitors

pcDNA6 MCV STco (codon optimised) was a gift from Patrick Moore (Addgene plasmid #40201; http://n2t.net/addgene:40201; RRID:Addgene_40201). pcDNA3.1-Flag-PP4C and RL-PP4C-TDN-HA were gifts from Marilyn Goudrealt (University of Toronto, Canada) and Tse-Hua Tan (National Health Research Institute, Taiwan), respectively. Primary antibodies targeting P-ERK1/2, ERK1/2 total, P-p38, p38 total, P-MK2, MK2 total, P-MSK1, MSK1 total, P-ATF2, ATF2 total, P-MKK4, MKK4 total and P-MKK3/6 were purchased from Cell Signalling Technologies and diluted 1 : 1000 in tris buffered saline (pH 7.6), containing 1% tween 20 (TBS-T) and 5% BSA, with the exception of P-ERK1/2 which was diluted 1 : 2000. GAPDH antibodies were purchased from Abcam and diluted 1 : 10 000 in TBS-T containing 5% non-fat milk. MCPyV ST was detected using 2T2 antibodies, kindly provided by Christopher Buck (NCI/CCR, Maryland, U.S.A.). Secondary antibodies conjugated to HRP were purchased from Dako (anti-mouse) and Cell Signaling Technologies (anti-rabbit) and diluted in TBS-T containing 5% non-fat milk 1 : 5000 and 1 : 2000, respectively. The p38 inhibitor SB202190 was purchased from Caltag Medsystems and used at 10 μM. SB202474 and U0126 were purchased from Cambridge Bioscience and used at 10 μM and 20 μM, respectively. All drugs were made up in DMSO at 1000× the concentration stated above.

### Cell lines

All HEK 293 cell lines were maintained in Dulbecco's modified Eagle's medium (DMEM) containing 10% (v/v) foetal bovine serum (FBS). HEK 293 cells were purchased from Thermo Fisher. HEK 293 cells were transfected using Lipofectamine 2000 (Life Technologies) at a ratio of 3 : 1 μl reagent : μg DNA. The inducible ST-flag HEK 293 cell line (i293-ST) was created using the HEK 293 FlpIn cell line purchased from Thermo Fisher and has been previously described [22,47]. The expression of MCPyV ST was induced in i293-ST cells through the addition of 2 μg/μl doxycycline hyclate (Dox). PeTa cells were maintained in RPMI 1640 containing 10% FBS and 1% penicillin/streptomycin [48].

### Western blotting

Cells were lysed using Leeds Lysis Buffer (25 mM glycerol phosphate, 20 mM tris, 150 mM NaCl, 1 mM EDTA, 1% (v/v) Triton X-100, 10% (v/v) glycerol, 50 mM sodium fluoride and 5 mM sodium pyrophosphate (pH 7.4)) supplemented with complete protease inhibitor cocktail (Roche) and protein phosphatase inhibitor cocktail 1 (Sigma), with incubation for 30 min on ice prior to 3 × 30 s on/off sonication before centrifugation (12 000 ***g***, 10 min, 4 °C) to pellet insoluble debris. Supernatants were aspirated and Pierce BCA assays (Thermo Fisher) performed to quantify the protein concentration of each sample. Equivalent protein mass for each sample was then separated by SDS–PAGE electrophoresis before transfer onto 0.45 μm nitrocellulose using a Bio-Rad Trans-blot Turbo transfer machine. Membranes were blocked in TBS-T containing 5% non-fat milk for 1 h at room temperature prior to 3× washes with TBS-T and overnight incubation at 4 °C in appropriately diluted primary antibodies. Membranes were then washed 3× with TBS-T before chemiluminescent detection using ECL Western Blotting Substrate (Promega) and Amersham Hyperfilm (GE Healthcare). Films were developed using an Xograph Compact X4 (Xograph Healthcare). Membranes were then stripped of antigen using a Blot Restore Kit (Merck) and subsequently reprobed with specific antibodies to detect total endogenous protein level and/or GAPDH to confirm equal loading.

### Cell proliferation and viability assays

Proliferation and viability of cells were assessed using the CellTiter 96® AQ_ueous_ One Solution Cell Proliferation Assay (Promega) as previously described [49]. Concurrent with experimentation, MTS reagent was added to cells following a 24, 48 or 72 h incubation with inhibitors.

### Gene expression data analysis

Two sets of expression data of MCC patient samples and primary normal human epidermal keratinocytes (NHEK) were selected and downloaded from gene expression omnibus (GEO): GSE39612 [6] and GSE29587. The sample compositions were as follows: GSE39612 (6 MCPyV-negative metastatic, 4 MCPyV-positive metastatic, 8 MCPyV-negative primary and 9 MCPyV-positive primary, profiled using Affymetrix Human Genome U144 Plus 2.0 Array) and GSE29587 (3 SB203580-treated (p38 inhibitor) and 3 untreated, profiled using Affymetrix Human Genome U133 Plus 2.0 Array). Differential expression analyses between different groups were performed using limma R package [50]. Differentially expressed genes were defined at adjusted *P*-value ≤ 0.05.

### Scratch assays

Scratch assays were performed using the i293-ST cell line. Cells were seeded in 24 well plates so that 24 h after induction, through the addition of DMEM containing 2 μg/ml Dox, confluency was at 100%. Scratches were then performed using a 50 μl ClipTip pipette tip (Thermo Fisher) and wells were washed with PBS to remove any dislodged cells and debris. Fresh DMEM containing 2 μg/ml Dox and DMSO vehicle control or 10 μM SB202190 was then added to the well. Images 1 and 48 h post-scratch were taken using an EVOS II microscope (Thermo Fisher). Average distance wound closure of *n *= 3 was calculated using ImageJ.

### Transwell migration assays

Transwell migration assays were performed using the CytoSelect 24-well Haptotaxis Assay and the MCPyV-positive MCC PeTa cell line. Briefly, 1 × 10^6 ^cells/ml were resuspended in serum depleted (0.5% (v/v) FBS) medium containing inhibitor or vehicle control and incubated for 24 h. 300 µl medium containing inhibitor and cells was added to the upper chamber and 500 µl of serum enriched (20% (v/v) FBS) growth medium was added to the lower chamber of the CytoSelect plate before incubation for 48 h. Cells in each chamber were resuspended by pipetting and aliquots mixed with trypan blue before quantification of cell number using a Countess II FL Automated Counter (Thermo Fisher). The percentage of migrated cells for each condition was calculated before comparison to an untreated control.

## Results

### MCPyV ST expression activates p38 MAPK signalling

The dysregulation of cell signalling is synonymous with cancer [51,52]. The association of MCPyV with MCC is well documented, however, despite evidence that p38 is active, functional studies have not been performed to identify phenotypic consequences [53]. We, therefore, investigated whether the expression of the major viral oncogene MCPyV ST alters p38 MAPK signalling.

**Figure 1.**
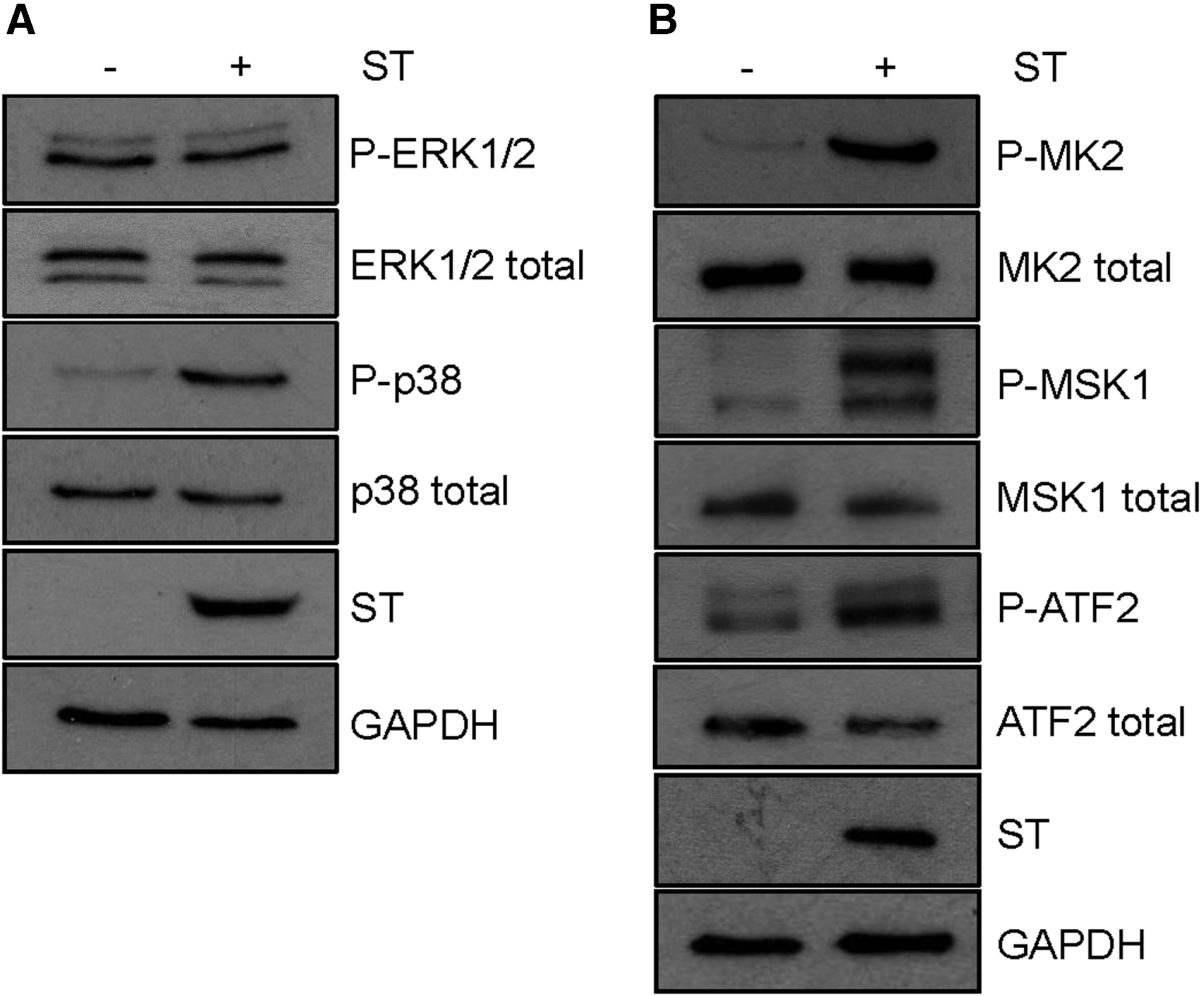
Expression of MCPyV ST activates the p38 MAPK pathway. (**A**) HEK 293 cells were transfected with vectors that express MCPyV ST or empty vector for 48 h. Following immunoblotting, antibodies were used to detect phosphorylated or total ERK1/2 and p38. ST specific antibodies (2T2) were used to detect MCPyV ST and antibodies specific for GAPDH used as a loading control. (**B**) Lysates were immunoblotted to detect phosphorylated and total MK2, MSK1 and ATF2, with 2T2 and GAPDH used to confirm MCPyV ST expression and equal loading, respectively. Blots are representative of *n *= 3.

Following overexpression of MCPyV ST in HEK 293 cells the phosphorylation of p38 MAPK markedly increased, with no change in total p38 MAPK expression (Figure 1A). No such changes in ERK1/2 phosphorylation were observed, consistent with previous studies [53]. To confirm that increased phosphorylation was associated with increased p38 activity, we investigated the phosphorylation of a range of downstream p38 MAPK targets (Figure 1B). MCPyV ST overexpression led to an increase in the phosphorylation of ATF2, MSK1 and MK2, confirming its ability to activate p38 MAPK signalling.

### MCPyV ST activation of MAPK pathway substrates is p38 specific

**Figure 2.**
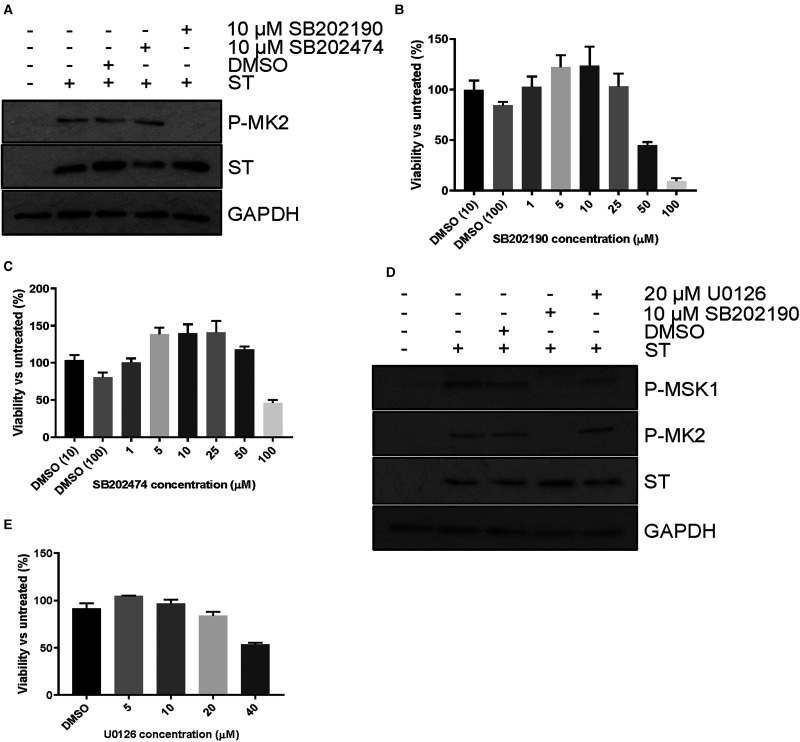
MCPyV ST activation of p38 is essential for downstream activation and independent of ERK. (**A**) HEK 293 cells were transfected with vectors that express MCPyV ST or empty vector for 48 h, with the addition of 10 μM SB202190 and 10 μM SB202474 for the final 24 h before lysis. Following immunoblotting antibodies were used to detect phosphoylated MK2, ST specific antibodies (2T2) were used to detect MCPyV ST and antibodies specific for GAPDH used as a loading control. (**B**,**C**) MTS viability assays were performed with a range of SB202190 and SB202474 concentrations to determine potential toxicity. DMSO (10) and (100) refer to an equivalent solvent volume present for 10 and 100 μM inhibitor, respectively. (**D**) HEK 293 cells were transfected with vectors that express MCPyV ST or empty vector for 48 h, with the addition of 10 μM SB202190 and 20 μM U0126 for the final 24 h before lysis. Following immunoblotting antibodies were used to detect phosphoylated MSK1 and MK2, ST specific antibodies (2T2) were used to detect MCPyV ST and antibodies specific for GAPDH used as a loading control. (**E**) MTS viability assays were performed with a range of U0126 concentrations to determine potential toxicity, with DMSO referring to an equivalent solvent volume present in 40 μM inhibitor. Immunoblots are representative of experimental *n *= 3.

Four p38 MAPKs are expressed in mammals: α, β, γ and δ. Cross-talk and redundancy across MAPKs is common [54], but MK2 is specifically activated by p38 MAPK isoform α. To confirm MK2 activation in response to MCPyV ST, cells expressing MCPyV ST were treated with SB202190, a selective inhibitor of p38α and p38β, alongside the inactive structural analogue SB202474 that possesses no inhibitory effect (Figure 2A). We found that treatment with SB202190 completely ablated MK2 phosphorylation, suggesting that MCPyV ST-mediated activation was p38 MAPK isoform α/β specific. Treatment with SB202474 did not influence the MCPyV ST induced hyperphosphorylation of MK2. Both SB202190 and SB202474 were also non-toxic at the concentrations assessed (Figure 2B,C).

Whilst MK2 is not unique in having only one upstream activator, the majority of MAPK substrates may be activated by more than one kinase. One such substrate is MSK1, which can be activated by both p38MAPK and ERK1/2 [54]. Despite no changes in ERK1/2 phosphorylation following MCPyV ST expression, we investigated its potential role through treatment with the MEK1/2 inhibitor, U0126. We found that MEK1/2 inhibition did not restrict MSK1 or MK2 phosphorylation, confirming that their activation was p38 MAPK specific (Figure 2D). Treatment with U0126 had no detrimental effects upon cell viability at the concentration used for assays (Figure 2E).

### MCPyV-positive MCC exhibit activated p38 MAPK signalling

**Figure 3.**
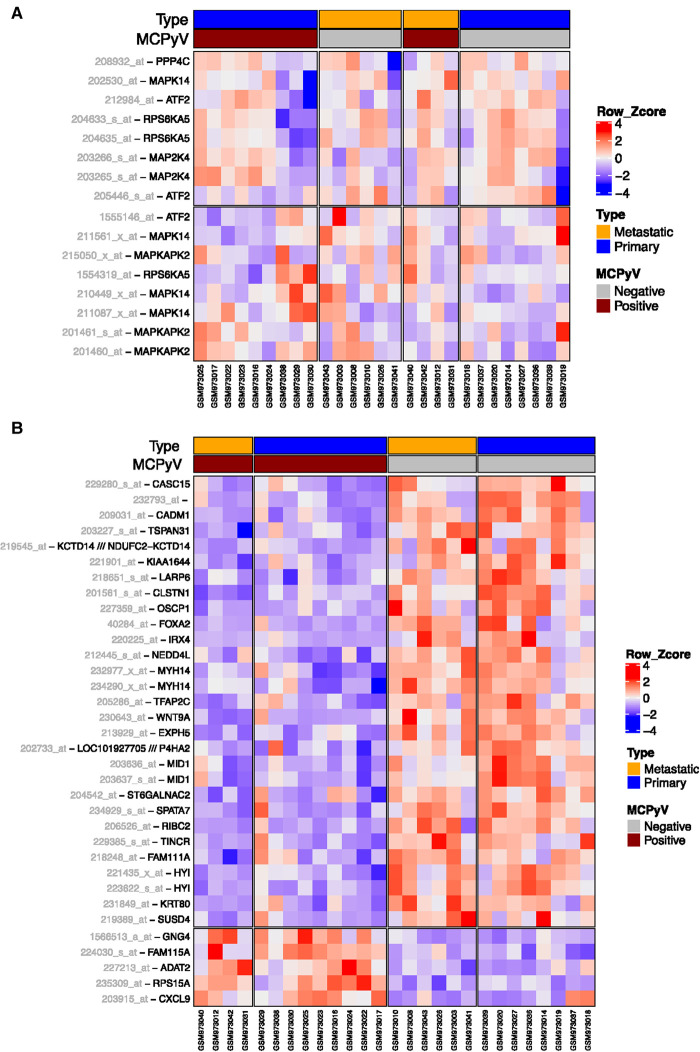
Expression profiles of p38 signalling genes. (**A**) The MCC dataset (GSE39612) was analysed and an expression heatmap generated for five p38 upstream genes across MCPyV-negative and MCPyV-positive MCC samples. (**B**) Downstream targets of p38 were identified via analysis of differentially expressed genes following inhibition of p38 signalling with SB203580 (GSE29587). The expression of these p38 downstream targets was subsequently analysed in the MCC dataset (GSE39612) and a heatmap generated across MCPyV-negative and MCPyV-positive MCC samples, showing probes that were inversely regulated compared with their expression following p38 inhibition in GSE29587. Within the heatmaps, red colour indicates high expression and blue indicates low expression.

To investigate if MCPyV ST-mediated activation of p38 MAPK signalling manifests clinically in MCPyV-positive tumours, we analysed publicly available gene expression profiles of MCC samples (GSE39612 [6]). In agreement with our *in vitro* assays (Figure 1A,B), the expression profile of p38 and downstream targets ATF2, MSK1 and MK2 were unaltered between MCPyV-negative and MCPyV-positive MCCs (Figure 3A).

To determine if MCPyV-positive MCC samples harbour increased p38 MAPK signalling, we initially analysed published data (GSE29587) which utilised the potent p38 MAPK-inhibitor, SB203580, to characterise the differential gene expression changes that occur due to inactivation of p38α and p38β signalling. We then cross-referenced p38 MAPK signalling-responsive in MCC samples, to establish if MCPyV-positive tumours possess a p38 MAPK signalling ‘signature’, compared with virus-negative MCC samples. Strikingly, 34 probes associated with 31 p38 MAPK-responsive genes showed significant MCPyV dependent expression differences that were inversely correlated with p38 MAPK inhibitor data (Figure 3B). This included the p38 MAPK target MID1, which is a negative regulator of basal and cytokine-induced p38 activation and LARP6, which is known to regulate microtubule development and cytokine activation of p38 signalling [55]. Together, these data provide evidence that MCPyV alters p38 signalling in the development and progression of MCC.

### Inhibition of p38 abrogates MCPyV ST induced cell migration

**Figure 4.**
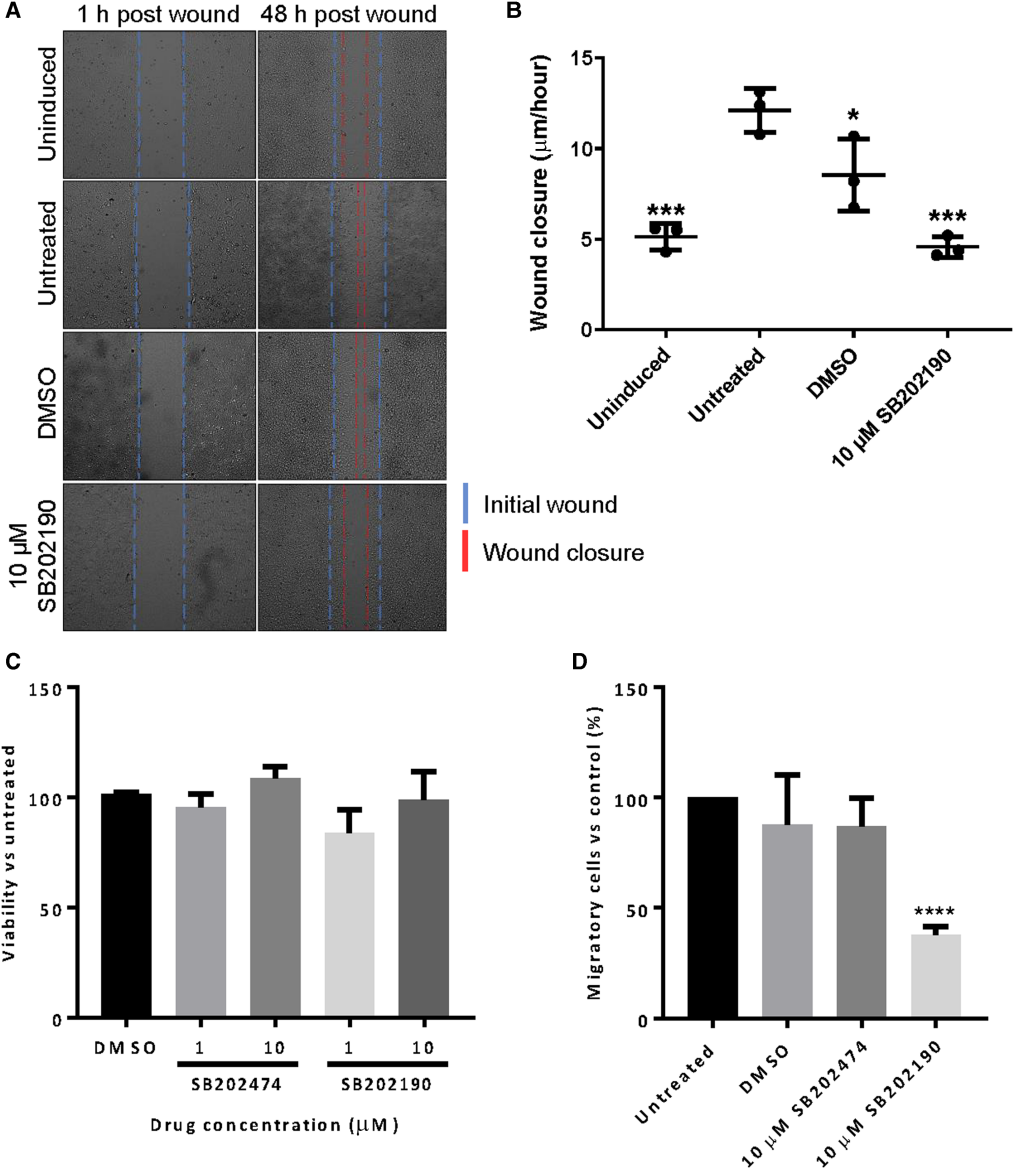
Inhibition of p38 leads to loss of MCPyV ST induced cell migration. (**A**) i293-ST cells were induced prior to a scratch-wound. Cells were treated with vehicle control and 10 μM SB202190 to evaluate inhibitor effects upon wound closure. Wells were imaged using an EVOS II microscope 1 and 48 h post wound. (**B**) Wound closure rate (μm/h) of *n *= 3 was calculated using ImageJ software. Each point represents the average wound closure of each experimental repeat. (**C**) MTS viability assays were performed using 1 and 10 µM SB202474 and SB202190 to determine inhibitor effects upon viability and proliferation of the MCPyV-positive PeTa cell line. DMSO contained a comparable volume of solvent present in 10 μM inhibitor. (**D**) Transwell assays were performed to determine the effect of 10 µM SB202474 and SB202190 upon the migratory potential of PeTa cells. Cells were incubated with inhibitor for 24 h before addition to transwell plates. The percentage of migrated cells relative to untreated control was evaluated using a Countess II FL Automated Counter following a 48 h incubation. Significance values presented in (**B**) and (**D**) were calculated using a one-way ANOVA, with multiple comparisons to untreated displayed, where *P < 0.05, ***P < 0.0005, ****P < 0.0001. Error bars show the standard deviation of three experimental repeats.

MCPyV ST induces cell motility and drives migration through cytoskeletal rearrangements that likely contribute to the highly metastatic phenotypes of MCPyV-positive MCCs [22,47]. MCPyV ST is known to promote the destabilisation of microtubules and enhance ADAM sheddase expression, enhancing degradation of the extracellular matrix and subsequent cell dissociation [22,23]. ST is also responsible for RhoA-GTPase-induced actin remodelling, leading to the formation of migratory F-actin-based filopodia. To determine whether p38 MAPK mediates the effects of MCPyV ST on cell motility, scratch assays were performed using i293-ST cells [47]. Expression of MCPyV ST in i293-ST cells was induced for 24 h prior to treatment with 10 μM SB202474 or 10 μM SB202190 and scratch-wounding. Cells were imaged at 1 and 48 h post-scratch and the average wound closure distance/hour was calculated (Figure 4A,B). Consistent with previous studies, i293-ST cells displayed significantly increased wound closure compared with uninduced cells. Interestingly, the treatment of i293-ST cells with SB202190 led to a recovery of wound closure rates that were comparable to uninduced cells, demonstrating that the inhibition of p38 MAPK abrogated MCPyV ST induced wound closure.

To further validate the importance of p38 MAPK activity to the pro-metastatic effects of MCPyV ST, MCPyV-positive MCC cells (PeTa) were treated with SB202190 or SB202474 and their effects on cell migration were assessed. Treatment with either inhibitor had no effect upon the proliferation of PeTa cells at both low (1 µM) or high (10 μM) concentrations (Figure 4C). Consistent with scratch assay results, 10 μM SB202474 did not influence the migration of PeTa cells in Transwell assays whilst treatment with 10 μM SB202190 potently inhibited cell migration, confirming that p38 MAPK activity was essential to MCPyV-ST induced cell motility in MCC cell lines (Figure 4D).

### MCPyV ST activation of p38 is MKK4 dependent, but independent of extracellular stimuli

**Figure 5.**
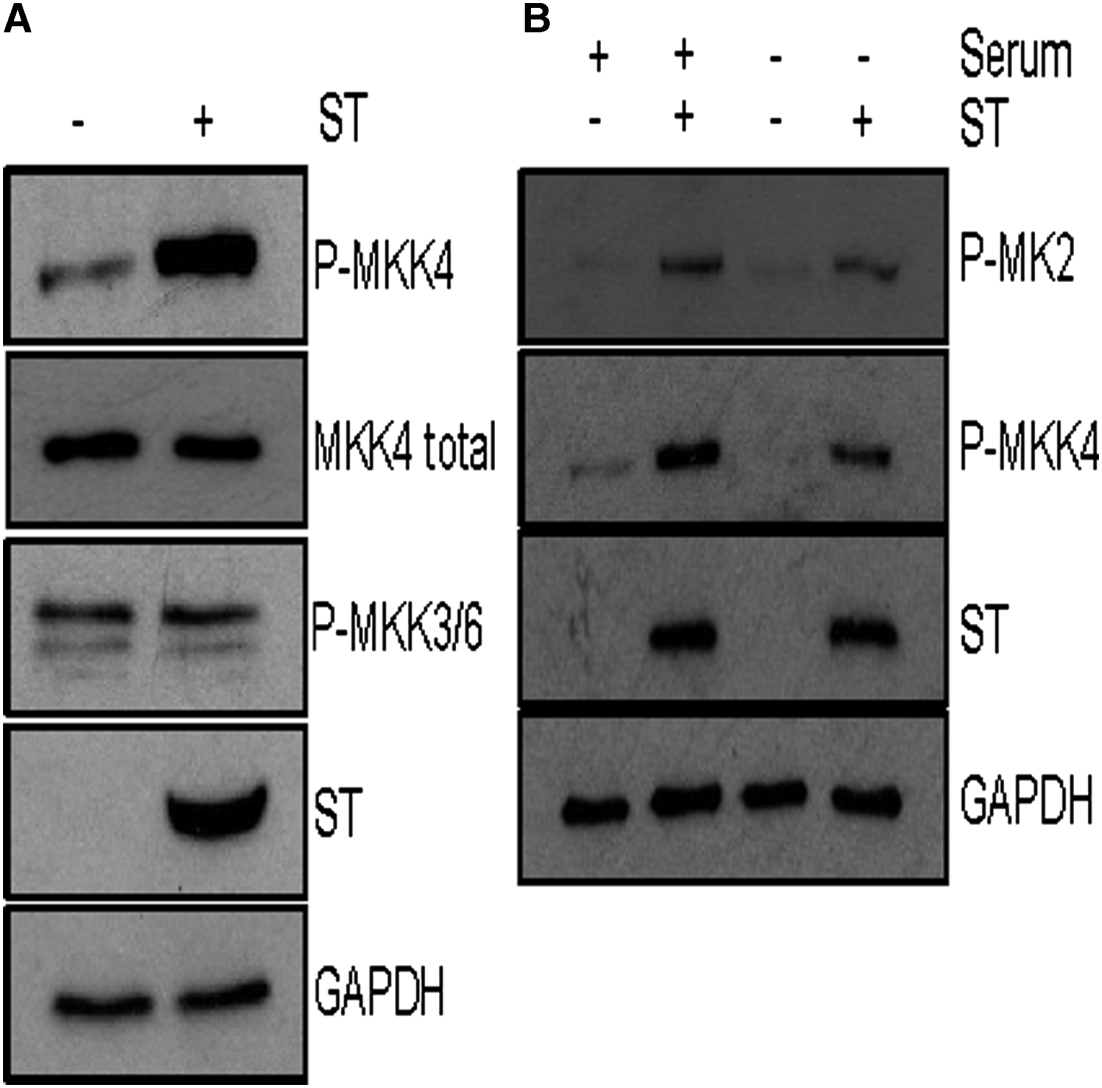
MCPyV ST induced activation of p38 is via MKK4 and is independent of extracellular stimuli. (**A**) HEK 293 cells were transfected with vectors that express MCPyV ST or empty vector for 48 h. Immunoblotting was performed to observe phosphorylation of MKK4 and MKK3/6. ST specific antibodies (2T2) were used to detect MCPyV ST and antibodies specific for GAPDH and endogenous levels of MKK4 used as loading controls. (**B**) HEK 293 cells were transfected with vectors that express MCPyV ST or empty vector for 48 h, with serum starvation or 10% (v/v) FBS containing DMEM applied for the final 2 h. Immunoblotting was performed to observe the effect of serum starvation upon phosphorylation of MK2 and MKK4. ST specific antibodies (2T2) were used to detect MCPyV ST and antibodies specific for GAPDH used as a loading control. Immunoblots are representative of experimental *n *= 3.

Following the identification that p38 MAPK is required for MCPyV ST induced cell motility, the mechanisms of MCPyV ST-mediated p38 MAPK activation were investigated. Canonically, p38 MAPK is activated by MKK3/6, however, MKK4 mediated activation has also been described. To determine whether MCPyV ST expression activated the pathways upstream of p38 MAPK, MKK3/6 and/or MKK4 phosphorylation were assessed following MCPyV ST expression (Figure 5A). Intriguingly, MKK4 displayed increased phosphorylation in cells overexpressing MCPyV ST, whilst MKK3/6 showed no increased phosphorylation in comparison with mock-transfected controls. These data strongly suggested that MCPyV ST-mediated p38 MAPK activation occurred through MKK4 as opposed to canonical MKK3/6 signalling.

MAPKs are activated by extracellular stress stimuli including UV exposure, mitogens and inflammatory cytokines [27]. To evaluate the role of exogenous mediators on p38 MAPK activation, mock or MCPyV ST expressing cells were incubated for 2 h in the presence or absence of serum (Figure 5B). Results indicated that MCPyV ST expression led to the phosphorylation of MK2 and MKK4 in serum-free cells. Additionally in mock-transfected cells, the basal phosphorylation of MKK4 decreased in response to serum starvation. These data suggest that MCPyV ST activates p38 MAPK signalling, independently of extracellular stimuli.

### Interaction of MCPyV ST with PP4C is essential for its effects on p38 MAPK signalling

**Figure 6.**
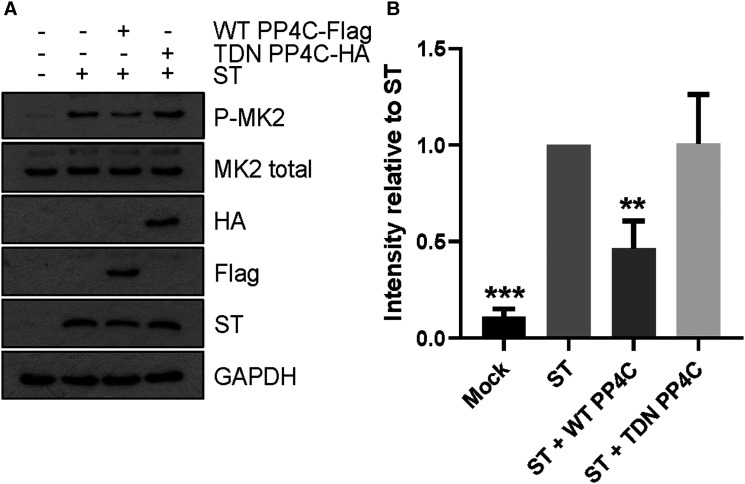
MCPyV ST interacts with PP4C to perturb pathway dephosphorylation. (**A**) HEK 293 cells were cotransfected with vectors that express MCPyV ST and WT PP4C-Flag or TDN PP4C-HA for 48 h. For sole expression of MCPyV ST, an empty vector of equivalent mass to PP4C vectors was included during transfection. Immunoblotting was performed using specific antibodies to determine phosphorylation and endogenous levels of MK2. Specific antibodies targeting Flag and HA tags were used to detect WT and TDN PP4C, respectively. ST specific antibodies (2T2) were used to detect MCPyV ST and antibodies specific for GAPDH used as a loading control. (**B**) Evaluation of MK2 phosphorylation by densitometry, normalised to total MK2 expression. Significance values were calculated using a one-way ANOVA, with multiple comparisons to ST displayed, where **P < 0.005 and ***P < 0.0005. Error bars show the standard deviation of three experimental repeats.

As MCPyV ST activates p38 MAPK through intrinsic methods, we assessed its interaction with cellular partners that are known to regulate p38 MAPK signalling. MCPyV ST interacts with cellular protein phosphatases including PP2A Aα and PP2A Aβ, but these are not essential for MCPyV ST induced cell motility [47]. Interestingly, MCPyV ST binding of PP4C is essential for microtubule destabilisation and actin remodelling, with PP4C-mediated dephosphorylation of the microtubule-associated protein stathmin, essential for enhanced cellular motility [22,47]. To investigate the role of PP4C in p38 MAPK activation, we co-overexpressed WT and transdominant negative (TDN) PP4C constructs with MCPyV ST and investigated their effects on p38 MAPK activation (Figure 6A,B). Results showed that the overexpression of WT-PP4C attenuated MK2 phosphorylation following MCPyV expression, whilst TDN PP4C had minimal effects. This suggested that the MCPyV ST interaction with PP4C prevents the dephosphorylation of p38 MAPK signalling components. This highlights PP4C as a key interaction that can restrict the metastatic potential of MCPyV-positive MCC.

## Discussion

Several oncoviruses such as human papillomavirus 16, Epstein–Barr virus, hepatitis B virus and SV40 dysregulate cell signalling pathways that contribute to metastasis [56–59]. MCPyV ST is a key driver of MCC that was recently reported to induce microtubule destabilisation, actin remodelling and cellular sheddase expression to stimulate pro-migratory phenotypes, all of which are synonymous with cancer metastasis [22,23,47].

We recently demonstrated that MCPyV ST induced cellular motility is in part, due to its interaction with PP4C, and that the activation of the Rho-GTPases cdc42 and RhoA are required. However, the molecular mechanisms governing these effects were not identified [47]. Here, we present a mechanism through which MCPyV ST enhances metastatic phenotypes. We show that MCPyV ST expression leads to the activation of p38 MAPK and its downstream targets including MK2, MSK1 and ATF2, consistent with previous studies that have identified a correlation between phosphorylated p38 MAPK and a poor MCC prognosis [53]. Consistent with previous findings [53], we observed no effects of MCPyV ST on the phosphorylation status of ERK1/2, suggesting that ST-mediated p38 MAPK activation is independent of other MAPK pathways.

MK2 is a unique target of p38α, which upon activation is translocated from the nucleus to the cytoplasm to initiate actin polymerisation via the phosphorylation and activation of heat shock protein 27 (HSP27) [60]. The dynamic and rapid polymerisation of globular monomeric actin (G-actin) into a filamentous form (F-actin) initiates morphological changes within the cell, priming it for migration [42]. Subsequent actin filaments form lamellipodia, filopodia or stress fibres that are dependent on the presence of actin-associated proteins that either enhance or perturb filament bundling [26,61]. In a motile cell, filaments bundle to form stress fibres that become polarised towards the leading edge, with retrograde recycling of the trailing edge of actin required to drive cell migration [62].

In this study, we show that the MCPyV ST enhancement of motility requires p38 MAPK activation, the inhibition of which ablates MCPyV ST induced cell migration. Whilst effective therapeutics to target p38 MAPK are lacking, pharmaceutical interest in this pathway remains given its dysregulation in a range of disease states. As such, targeting p38 MAPK may offer a potential route to reduce the metastatic potential of MCC.

**Figure 7.**
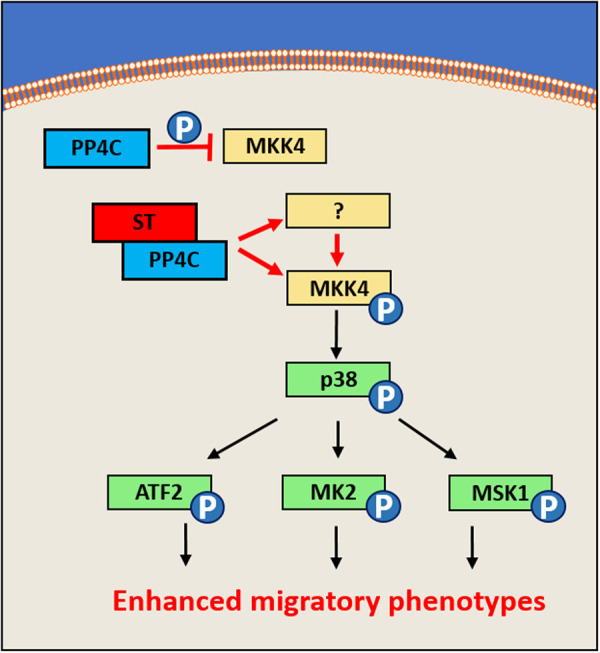
Proposed mechanism via which MCPyV ST activated the p38 pathway to regulate actin dynamics. MCPyV ST interacts with PP4C to dysregulate its role in regulating the phosphorylation of either MKK4 or an upstream kinase. Following MCPyV ST binding of PP4C, MKK4 activation, in turn, phosphorylates p38, which in turn phosphorylates substrates including MK2, ATF2 and MSK1. The activation of downstream targets of p38 subsequently enhance migratory phenotypes associated with MCPyV ST.

We further show that the activation of p38 MAPK is independent of extracellular stimuli and via non-canonical MKK4 signalling, as opposed to well-characterised MKK3/6 mediators. Intriguingly, we report that loss of MCPyV ST interaction with PP4C led to reduced phosphorylation of MK2, in agreement with previous data showing that actin remodelling and filopodia formation rely upon ST-PP4C binding. We, therefore, propose a model whereby MCPyV ST interacts with PP4C to dysregulate MKK4 or an upstream kinase and ultimately p38 MAPK (Figure 7). Upstream of the four MAPKs is a complex network of kinases, which through sequential phosphorylation and activation ultimately lead to phosphorylation of proteins such as p38 MAPK. Whilst it was not investigated in this study, given previous evidence that Rho-GTPase activity is essential for MCPyV ST induced actin rearrangements and enhance motility, there is the potential that such protein families may be responsible for p38 MAPK activation, however, further work is required to map such pathways in more detail.

Downstream of MCPyV ST induced p38 MAPK activation a range of substrates are phosphorylated, with MK2 a prime candidate that facilitates the enhanced migratory phenotypes via an MK2-HSP27-F-actin axis. Importantly, the ST-p38 MAPK axis delinated by our *in vitro* studies is also present in MCPyV-positive MCC samples, with numerous p38 MAPK downstream targets differently expressed when compared with virus-negative MCC.

In summary, we provide evidence that MCPyV ST activates p38 MAPK through its interaction with PP4C to enhance cellular motility. Furthermore, the inhibition of p38 MAPK can abrogate cell motility and restrict highly metastatic MCC. Targeting p38 MAPK activity either directly or through upstream kinases such as MKK4, may, therefore, present a viable therapeutic target to restrict metastasis and secondary tumour formation, improving the prognosis of MCC patients.

## Abbreviations

DMEM: Dulbecco's modified Eagle's medium
FBS: foetal bovine serum
LT: large tumour antigen
MAPKs: mitogen-activated protein kinases
MCC: Merkel cell carcinoma
MCPyV: Merkel cell polyomavirus
ST: small tumour antigen
SV40: simian virus 40
TDN: transdominant negative
tLT: truncated LT
